# Characterization of inflammatory cell infiltrate of scleroderma skin: B cells and skin score progression

**DOI:** 10.1186/s13075-018-1569-0

**Published:** 2018-04-18

**Authors:** Silvia Bosello, Cristiana Angelucci, Gina Lama, Stefano Alivernini, Gabriella Proietti, Barbara Tolusso, Gigliola Sica, Elisa Gremese, Gianfranco Ferraccioli

**Affiliations:** 1 Unità Operativa Complessa di Reumatologia, Istituto di Reumatologia e Scienze Affini, Università Cattolica del Sacro Cuore, Rome, Italy; 2 Fondazione Policlinico Universitario Agostino Gemelli, Via G. Moscati, 31-00168 Rome, Italy; 3 Istituto di Istologia ed Embriologia, Università Cattolica del Sacro Cuore, Rome, Italy

**Keywords:** Systemic sclerosis, T cells, B cells, Macrophages, Skin involvement

## Abstract

**Background:**

The purpose of this study was to investigate the frequency and the distribution of inflammatory cell infiltrate in two sets of cutaneous biopsies derived from clinically affected and unaffected skin in patients with systemic sclerosis (SSc) and to test correlation between the cell infiltrate and the progression of skin involvement.

**Methods:**

Skin was immunohistochemically assessed to identify CD68, CD3, CD20 and CD138-positive (+) cells in clinically affected and unaffected skin in 28 patients with SSc. Patients were followed for 6 months and the characteristics of the infiltrate were analyzed according to disease duration, clinical features and skin involvement progression.

**Results:**

In all SSc cutaneous specimens, cellular infiltrates were found in a perivascular location predominantly in the mid and deeper portions of the dermis. All the analyzed biopsies showed a CD3^+^ and CD68^+^ cell infiltrate and the mean number of CD3^+^ and of CD68^+^ cells was higher in clinically involved skin (CD3^+^, 71.7 ± 34.6 and CD68^+^, 26.3 ± 8.4, respectively) than in clinically uninvolved skin (CD3^+^, 45.7 ± 36.0 and CD68^+^, 13.6 ± 6.1, respectively) (*p* < 0.001 for both comparisons). CD20^+^ cells were found in 17 (60.7%) patients and in these patients the mean number of CD20^+^ cells was higher in clinically involved (4.7 ± 5.9) than in uninvolved skin (1.9 ± 2.9), (*p* = 0.04). There was a greater number of CD20^+^ cells in patients with early SSc compared with patients with long-standing disease. CD138^+^ cells were found in 100% of biopsies of clinically involved skin and in 89.3% of biopsies of uninvolved skin. The mean number of CD138^+^ cells was higher in clinically involved skin (3.6 ± 2.3) than in clinically uninvolved skin (1.9 ± 1.7), (*p <* 0.001). Seven patients experienced more than 20% worsening in the skin score after 6 months of follow up; all of them had a CD20^+^ skin infiltrate on biopsy of clinically involved skin.

**Conclusions:**

Our results confirm that mononuclear cells are present in the skin of all patients with SSc, underlining the role of inflammatory cell infiltrates in skin involvement in SSc. B cells in the skin seem to characterize patients with early diffuse skin disease and to correlate with skin progression.

**Electronic supplementary material:**

The online version of this article (10.1186/s13075-018-1569-0) contains supplementary material, which is available to authorized users.

## Background

Progressive systemic sclerosis (SSc) or scleroderma is a chronic connective tissue disorder characterized by vascular and cellular abnormalities that predominate in the early stages of disease and eventually lead to extensive cutaneous and visceral fibrosis, which is most prominent in the later stages [1–3]. The pathologic phenotype of the disease is complex and, consequently, its etiology and the effective therapies to prevent its progression remain elusive.

Cutaneous mononuclear cells are a frequent histopathological finding in SSc. Previous studies have recorded an increase in the number of mast cells [4, 5], macrophages [3, 6–11] and T lymphocytes [3, 6, 8–12], in particular in the early stages of SSc. Furthermore, microarray analysis in the lungs and in the skin of patients with scleroderma indicates overexpression of macrophage marker genes [13–16]. In vitro studies have demonstrated that all these cells can produce several cytokines and stimulate fibroblasts [1].

To date, although there have been various reports of studies of the B cell infiltrate in SSc [3, 6, 8–10, 13, 17–19], its characterization in skin affected by scleroderma is still incomplete. Recently, some encouraging results suggested a possible use of an anti-CD20 monoclonal antibody (rituximab) for the treatment of early, progressive and diffuse scleroderma, suggesting a role for B cells in the pathogenesis of the disease in the early stage [17–23]. In fact, it has been shown that patients with SSc present with autoantibody production [24], hyper-γ-globulinemia, polyclonal B cell hyperactivity [25] and abnormalities in the B cell compartments, characterized by an increase in naïve cells and a decrease in activated memory B cells [26]. In addition, in scleroderma there is markedly increased expression of CD19, a signal transduction molecule of B cells that regulates production of autoantibodies, in both memory and naïve B cells [26, 27]. In patients with diffuse SSc (dSSc), analysis of DNA microarrays of cutaneous biopsies have demonstrated higher expression of clusters of genes of CD20^+^ cells and of plasma cells [13].

In an attempt to further clarify the characteristics of the cellular infiltrate, and mostly the possible role of B cells in skin fibrosis, we investigated the frequency and the distribution of mononuclear cells in two sets of cutaneous biopsies derived from clinically affected and unaffected skin from patients with SSc. The characteristics of the infiltrate were also analyzed according to disease duration, clinical features of the patients and skin score modification after 6 months.

## Methods

### Patients

Patients with SSc (n = 28 (24 female and 4 male)) attending the outpatient clinic of the Division of Rheumatology of our institution agreed to undergo skin biopsies on both clinically affected and unaffected skin and were included in the study. All patients fulfilled the old and the new classification criteria for scleroderma proposed by the European League Against Rheumatism (EULAR) and the American College of Rheumatology (ACR) [28, 29]. Informed signed consent to undergo biopsies and to provide skin samples and clinical data for research purposes was provided by all the patients. This research has been performed in accordance with the Declaration of Helsinki and it was approved by our institutional ethics committee (Comitato Etico Università Cattolica del Sacro Cuore 1883/12).

Demographic and clinical characteristics were collected in all patients with SSc enrolled in the study. Patients were grouped according to the classification proposed by LeRoy in patients with limited SSc (lSSc) or diffuse SSc (dSSc) [30]. The extent of skin involvement was evaluated by the Rodnan skin score, performed by two assessors (always the same at every evaluation) whose results were averaged [31], at the time of skin biopsy and after a mean follow-up time of 6.5 ± 0.8 months. A modification of skin score higher than 20% was considered clinically significant progression [32, 33]. Antinuclear antibodies (ANA) were determined by indirect immunofluorescence using Hep-2 cells as substrates and autoantibody specificities were further assessed by ELISA (Shield, Dundee, UK).

Cutaneous specimens were taken from patients with SSc, by surgical excision with a 6-mm punch from the distal forearm for the clinically involved skin (skin score >1 at this site) and from the buttock for clinically uninvolved skin (skin score = 0 at this site). The presence of mononuclear inflammatory cells was investigated in all skin specimens, in particular CD3 as a marker of T lymphocytes, CD20 as a marker of B-lymphocytes (local mature B cells and memory B cells), CD138 as a marker of plasma cells and CD68 as a marker of residential macrophages.

At the time of the skin biopsies, the activity index [34] and the severity index [35] were assessed and Global Health (GH) status and Health Assessment Questionnaires (HAQ) were administered to patients to evaluate the influence of the disease on daily functions.

All 28 patients continued to receive iloprost (an infusion of 0.5–2 ng/kg body weight/min for 5 days every 2 months), calcium channel blockers (nifedipine 20–40 mg/day) and acetylsalicylic-acid from the moment of diagnosis. Patients receiving corticosteroids or immunosuppressive drugs at the time of skin biopsy were excluded from the study. During the follow-up period, 12 patients were treated with anti-CD20 monoclonal antibody (rituximab) with or without cyclophosphamide for skin disease progression and/or lung involvement, while the other 16 patients did not receive immunosuppressive drugs.

Internal organ involvement was evaluated no longer than one month before or after the skin biopsies. All patients with SSc underwent pulmonary function tests (PFTs) to define forced vital capacity (FVC) and diffusing capacity for carbon monoxide (DLCO), and high resolution computed tomography (HRCT) was performed to assess lung involvement [36]. Renal involvement was defined as a scleroderma crisis or as the presence of proteinuria or elevation in creatinine serum level. Electrocardiography (ECG) and echocardiography were also performed in all patients: cardiac involvement was defined as the presence of conduction disturbance, left ventricular ejection fraction (LVEF) and <50%, pulmonary artery systolic pressure (PASP) >35 mmHg on echocardiography. Gastro-intestinal involvement was defined as the presence of gastroesophageal reflux symptoms or the evidence of gastrointestinal motility disturbance.

Four female healthy subjects (age range 36–55 years) gave their informed consent to undergo forearm skin biopsy, as controls.

### Immunohistochemical analysis

Immunohistochemical analysis was carried out on 5-μm thick tissue sections on polylysine-coated slides. After deparaffinization and rehydration, antigen retrieval was performed. Slide-mounted sections were heated in a microwave oven at 700 watt two times for 4 min in 10 mmol/L sodium citrate buffer (pH 6.0). Quenching of endogenous peroxidase activity was performed with Tris-buffered saline (TBS) (pH 7.6) containing 2% hydrogen peroxide (H_2_O_2_) for 10 min at room temperature (RT). To prevent non-specific binding, blocking was performed with Super block (UCS Diagnostics, Rome, Italy) for 8 min at RT. Sections were incubated with anti-CD3 mouse monoclonal antibodies (mAb) (1:50 dilution; Clone PS1, Abcam, Cambridge, MA, USA) or anti-CD20 mouse mAb (1:100 dilution; Clone L26; Novus Biologicals, Littleton, CO, USA) or human anti-CD138 (Syndecan-1) rabbit polyclonal antibodies (Ab) (1:25 dilution; Spring Bioscience, CA, USA). Tissues were then incubated with the Super Picture HRP Polymer Conjugated Broad Spectrum (Invitrogen, Carlsbad, CA, USA) for 30 min at RT and the chromogenic reaction was developed with 3,3′-diaminobenzidine tetrahydrochloride solution (Zymed Laboratories, South San Francisco, CA, USA). The nuclei were lightly counterstained with hematoxylin. Negative controls without primary Abs were performed for all reactions. Human tonsil specimens were used as positive controls. CD68 was immunohistochemically assessed using an autostainer (BOND MAX III, Leica Biosystems, Newcastle, UK), according to the manufacturer’s standard protocol, using an anti-CD68 mouse anti-human mAb (ready to use; clone 514H12, Leica Biosystems) [37].

Cellular infiltrates were studied in the dermis and/or subcutaneous tissue and were classified as either perivascular or diffuse. Positive cells were counted by two independent observers in a total of six randomly selected fields (total area 7.38 mm^2^) for each section at × 400 magnification, under a light microscope (Axioskop 2 plus, Zeiss). The total number of positive cells was calculated and reported as mean ± SD, median and range.

### Statistical analysis

All analyses were carried out using SPSS 15.0 (Chicago, IL, USA). Categorical variables were expressed as numbers, and quantitative variables as mean ± SD if normally distributed, and as median plus range if not. Categorical variables were analyzed using the chi-square (Χ^2^) test or Fisher’s test, depending on sample size restrictions. Non-normally distributed continuous data were compared using the Mann-Whitney test and the Wilcoxon test (for paired data). A value of *p* < 0.05 was considered statistically significant. Correlation was tested using Spearman’s rank order correlation for non-normally distributed interval data.

## Results

### Demographic, clinical and immunological characteristics of enrolled patients with SSc

Demographic and clinical characteristics of patients with SSc enrolled in the study are shown in Table 1.

**Table 1:** Demographic and clinical characteristics of patients with SSc enrolled in the study

Characteristic	Value
Age (years), mean (SD)	44.6 (15.4)
Age (years), median (range)	46.0 (20.0–67.0)
Female, number (%)	24 (85.7)
Male, number (%)	4 (14.3)
Disease duration (months), mean (SD)	44.7 (71.5)
Disease duration (months), median (range)	16.00 (3–360)
Early disease, number (%)	19 (67.9)
Long-standing disease, number (%)	9 (32.1)
Anti-Scl-70 positivity, number (%)	21 (75.0)
ACA positivity, number (%)	3 (10.7)
RNA polymerase III positivity, number (%)	1 (3.6)
ANA positivity^a^, number (%)	3 (10.7)
dSSc, number (%)	20 (71.4)
lSSc, number (%)	8 (28.6)
Skin score, mean (SD)	15.8 (11.3)
Skin score, median (range)	14.0 (2.0–43.0)
Activity index, mean (SD)	4.4 (2.0)
Activity index, median (range)	4.2 (1.0–8.5)
Severity index, mean (SD)	8.0 (2.6)
Severity index, median (range)	7.5 (4.0–14.0)
FVC (%), mean (SD)	87.8 ± 22.1
FVC (%), median (range)	94.0 (41.0–123.0)
DLCO (%), mean (SD)	57.3 ± 18.4
DLCO (%), median (range)	57.0 (26.0–93.0)
HRCT interstitial score, mean (SD)	7.6 (2.3)
HRCT interstitial score, median (range)	7.0 (4.0–14.0)
HRCT alveolar score, mean (SD)	6.7 (5.0)
HRCT alveolar score, median (range)	6.0 (0.0–16)
ESR, mm/h, mean (SD)	20.4 (20.3)
ESR, median (range)	12.0 (2.0–84.0)

The values are indicated as the mean (SD) and median (range) or number (percentage)

*ANA* antinuclear antibodies, *ACA* anticentromere antibodies, *anti-Scl-70* anti-topoisomerase antibodies, *dSSc* diffuse skin disease, *lSSc* limited skin disease, *FVC* forced vital capacity, *DLCO* diffusion lung carbon monoxide, *ESR* erythrocyte sedimentation rate

^a^ANA positivity: two patients who were ANA-positive presented with a homogeneous pattern, and one patient presented with a nucleolar pattern

The mean age (± SD) of the patients with SSc was 44.6 ± 15.4 years and the median disease duration was 16.0 (range 3.0–360.0) months. There were 19 patients (67.9%) with early disease, defined as diagnosis up to 3 years after the occurrence of Raynaud’s phenomenon; the remaining 9 patients (32.1%) had long-standing disease. There were 20 patients (71.4%) with dSSc. The baseline mean modified Rodnan skin score was 15.8 ± 11.3 (range 2.0–43.0). Anti-topoisomerase antibodies (anti-Scl-70 Abs) were present in 21 (75.0%) patients and anti-centromere Abs (ACA) in 3 patients (10.7%). One patient presented with RNA polymerase III autoantibody positivity; the other three patients were ANA positive only (one with a nucleolar pattern and two with a homogeneous pattern) (Additional file 1: Table S1).

### Skin CD20^+^ B-cells and CD138^+^ plasma cell infiltrates characterize patients with SSc based on disease duration and subset

In all 56 cutaneous specimens from patients with SSc, mononuclear cell infiltrates were found in a perivascular location, predominantly in the mid and deeper portions of the dermis. CD20^+^ cells were found in 17 (60.7%) out of the 28 patients with SSc: 9 of these patients (52.9%) had CD20^+^ cells in either clinically involved or uninvolved skin, 7 (41.2%) had CD20^+^ cells only in the involved skin and one patient with diffuse skin disease and anti-Scl-70 Abs had CD20^+^ cells only in clinically uninvolved skin. Importantly no CD20^+^ cells were found in biopsy specimens from healthy volunteers.

**Fig. 1:**
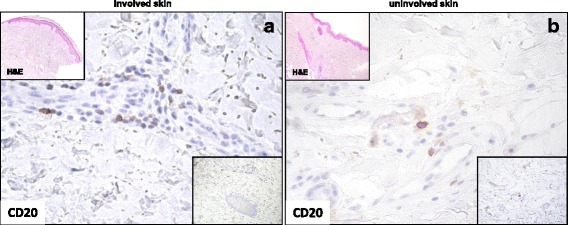
CD20 cell staining in involved and uninvolved skin from a patient with systemic sclerosis (SSc). **a** Cellular infiltrate in involved skin (distal forearm biopsy) from a patient with early anti-Scl-70^+^ diffuse SSc showing an appreciable number of CD20^+^ cells in perivascular areas. **b** Uninvolved skin (buttock biopsy) from the same patient showing the presence of a smaller number of CD20^+^ cells around blood vessels. Original magnification × 400 (**a** and **b**). Nuclei were counterstained by hematoxylin. Bottom insets show the infiltrating cells at a lower magnification (× 200). Insets at the top of the figures show hematoxylin and eosin (H&E) staining of involved and uninvolved skin (× 50)

In the subgroup that had CD20^+^ staining, the mean number of CD20^+^ cells was higher in involved (4.7 ± 5.9) than in uninvolved skin (1.9 ± 2.9), (*p* = 0.04, Table 2). Among the 17 patients with CD20^+^ cells on skin biopsy, 12 patients (70.6%) had early disease, 14 (82.3%) had diffuse skin involvement and 12 (70.6%) had anti-Scl-70 Ab positivity. Patients with early SSc had higher numbers of CD20^+^ cells (6.3 ± 6.5) than patients with long-standing disease (1.2 ± 0.9, (*p* = 0.009)) in involved skin. In clinically involved skin, patients with dSSc had numbers of CD20^+^ cells (4.9 ± 6.4) comparable to patients with lSSc (4.3 ± 4.0), but interestingly all patients with CD20^+^ cells in the clinically uninvolved skin had diffuse disease (Fig. 1).

**Table 2:** CD68^+^, CD3^+^, CD20^+^ and CD138^+^ cell counts on paired skin specimens in the 28 patients with SSc

	Clinically involved skin	Clinically uninvolved skin	*P*
CD68^+^ mean (SD)	26.3 (8.3)^a^	13.6 (6.1)^a^	0.001
CD68^+^ median (range)	24.5 (12.0–40.0)	12.5 (3.0–25.0)	
CD3^+^ mean (SD)	71.7 (34.6)^a^	45.7 (36.0)^a^	0.001
CD3^+^ median (range)	70.0 (7.0–146.0)	29.8 (1.0–130.0)	
CD20^+^ mean (SD)*	4.7 (5.9)^b^	1.9 (2.9)^b^	0.04
CD20^+^ median (range)	2.0 (0.0–25.0)	1.0 (0.0–11.5)	
CD138^+^ mean (SD)	3.6 (2.3)^a^	1.9 (1.7)^c^	< 0.001
CD138^+^ median (range)	3.0 (0.5–11.5)	1.5 (0.0–9.0)	

The values are the mean (SD) and median (range)

^a^Mean (SD) and median (range) of CD68^+^*,* CD3^+^, CD138^+^ in clinically involved skin (forearm) and of CD68^+^ *and* CD3^+^ in clinically uninvolved skin (buttock) refers to the duplicate skin samples from patients

^b^Mean (SD) and median (range) of CD20^+^ was calculated considering only the 17 patients (60.7%) that had almost one CD20+ cell in clinically involved skin or in clinically uninvolved skin

^**c**^Mean (SD) and median (range) of CD138^+^ in clinically uninvolved skin was calculated considering 25 out of 28 (89.3%) patients with CD138 + cells in uninvolved skin specimens

All patients with B cells in clinically uninvolved skin had anti-Scl-70 Abs, RNA polymerase III autoantibody positivity or ANA positivity; none of the patients with ACA positivity had a B cell aggregate in uninvolved skin.

The 17 patients with CD20^+^ cells in their skin had shorter disease duration (33.2 ± 36.8 months), compared to patients without B cell aggregate in skin biopsies (61.9 ± 104.6 months), but this difference was not statistically significant, and there was no significant correlation between the number of CD20^+^ cells and disease duration. No association emerged between the presence of CD20^+^ cells and restrictive lung involvement or heart or gastrointestinal disease.

The number of CD20^+^ cells in involved skin cells directly correlated with the number of CD20^+^ cells in uninvolved skin (*r* = 0.62, *p* < 0.001) and with the number of CD3^+^ cells in involved skin (*r* = 0.4, *p* = 0.03). Furthermore, CD20^+^ cells in involved skin were inversely correlated with DLCO values (*r* = − 0.5, *p* = 0.01) and with FVC values (*r* = − 0.5, *p* = 0.003).

CD138^+^ cells were found in 100% of involved skin biopsies and in 89.3% of clinically uninvolved skin samples (Table 2), but none of the healthy controls had plasma cells in the skin.

**Fig. 2:**
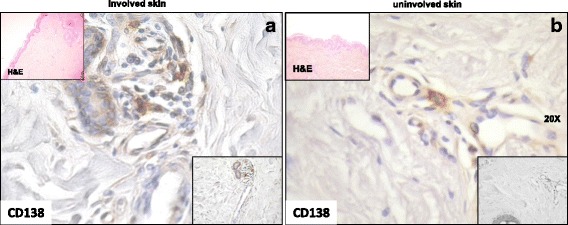
CD138 cell staining in involved and uninvolved skin from a patient with systemic sclerosis (SSc). **a** Involved skin (distal forearm biopsy) from a patient with an early anti-Scl-70^+^ diffuse SSc showing an appreciable number of CD138^+^ cells in perivascular areas. **b** Uninvolved skin (buttock biopsy) of the same patient showing the presence of a smaller number of CD138^+^ cells around blood vessels. Original magnification × 400 (**a** and **b**). Nuclei were counterstained by hematoxylin. Bottom insets show the infiltrating cells at a lower magnification (× 200). Insets at the top of the figures show hematoxylin and eosin (H&E) staining of involved and uninvolved skin (× 50)

The mean number of CD138^+^ cells was higher in clinically involved skin (3.6 ± 2.3) than in uninvolved skin (1.9 ± 1.7 (*p* < 0.001)) (Fig. 2). Although in patients with early disease the number of CD138^+^ cells in involved skin (4.2 ± 2.6) and in uninvolved skin (2.1 ± 1.9) was higher than in longstanding disease (involved skin 2.6 ± 1.4, uninvolved skin 1.7 ± 1.1), this difference was not statistically significant. Furthermore, no statistically significant difference was found in the mean number of CD138^+^ in patients with diffuse skin disease either in involved (4.1 ± 2.6) or uninvolved (2.1 ± 1.9) skin compared to patients with lSSc (involved skin 2.5 ± 1.0, uninvolved skin 1.7 ± 1.0). No differences were observed in plasma cell infiltrates in patients with different autoantibodies specificities or in patients with different organ involvement.

In involved skin, the number of CD138^+^ cells directly correlated with the number of CD20^+^ cells (*r* = 0.6, *p* = 0.01) and with the number of CD3^+^ cells (*r* = 0.5, *p* = 0.01), whereas there was no significant correlation on analysis of the non-affected skin samples.

### Skin CD3^+^ T-lymphocyte infiltrates are differentially presented in patients with SSc based on the skin compartment and autoantibody positivity

CD3^+^ cells were found in all skin biopsies of both healthy subjects and patients with SSc. In healthy subjects, CD3^+^ cell number was significantly smaller (8.0 ± 2.0) compared to that in involved (71.7 ± 34.6, (*p* < 0.001)) or uninvolved (45.7 ± 36.0, (*p* < 0.001)) skin from patients with SSc.

**Fig. 3:**
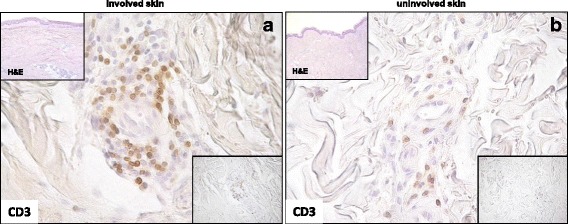
CD3 cell staining in involved and uninvolved skin from a patient with systemic sclerosis (SSc). **a** Involved skin (distal forearm biopsy) from a patient with early anti-Scl-70^+^ diffuse SSc showed massive infiltration with CD3^+^ cells in perivascular areas. **b** Uninvolved skin (buttock biopsy) from the same patient showing the presence of a smaller number of CD3^+^ cells around blood vessels. Original magnification × 400 (**a** and **b**). Nuclei were counterstained by hematoxylin. Bottom insets show the infiltrating cells at a lower magnification (× 200). Insets at the top of the figures show hematoxylin and eosin (H&E) staining of involved and uninvolved skin (× 50)

The mean number of CD3^+^ cells was higher in clinically involved skin than clinically uninvolved skin, (*p* = 0.001) (Table 2, Fig. 3). No statistically significant difference in the mean number of CD3^+^ cells was found in skin from patients with early compared to long-standing SSc, either in involved or uninvolved skin (Table 2).

The mean number of CD3^+^ cells in involved skin was higher in patients with dSSc (75.1 ± 35.8) than in patients with lSSc (63.2 ± 31.7), but this difference was not statistically significant, probably due to the wide variation in cell numbers.

The number of CD3^+^ cells was comparable in involved skin from patients with SSc regardless to anti-Scl-70 Ab positivity. In clinically uninvolved skin biopsies, patients with anti-Scl-70 Abs had a greater number of T lymphocytes (51.7 ± 38.7) than anti-Scl-70 Ab-negative patients (27.7 ± 18.8, (*p* = 0.05)). It is worth mentioning that the small group of patients with ACA positivity had a smaller number of CD3^+^ cells in both affected and unaffected skin. The number of CD3^+^ cells in involved skin directly correlated with the number of CD3^+^ cells in uninvolved skin (*r* = 0.5, *p* = 0.009) and inversely correlated with DLCO (*r* = − 0.4, *p* = 0.002), but there was no correlation with the Rodnan skin score, disease activity or severity scores.

### Skin CD68^+^ macrophage infiltrates are differentially presented in patients with SSc based on the skin compartment

**Fig. 4:**
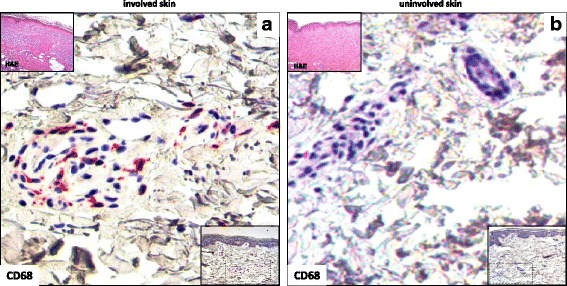
CD68 cell staining in involved and uninvolved skin from a patient with systemic sclerosis (SSc). **a** Involved skin (distal forearm biopsy) from a patient with early anti-Scl-70^+^ diffuse SSc showed an infiltrate of CD68^+^ cells (red) in perivascular areas. **b** Uninvolved skin (buttock biopsy) from the same patient showing the presence of a smaller number of CD68^+^ cells around blood vessels. Original magnification × 400 (**a** and **b**). Nuclei were counterstained by hematoxylin. Insets at the bottom of the figures show the infiltrating cells at a lower magnification (× 200). Insets at the top of the figures show hematoxylin and eosin (H&E) staining of involved and uninvolved skin (× 50)

Using paired skin samples, CD68^+^ cell count was significantly higher in clinically involved (26.3 ± 8.3) compared to uninvolved skin from patients with SSc (13.6 ± 6.1) (*p* = 0.001) (Fig. 4), the CD68^+^ cells having a preferential perivascular distribution within the dermis.

Grouping patients with SSc according to the disease subset, we found that CD68^+^ cell count was significantly higher in clinically uninvolved skin from patients with SSc with a diffuse phenotype (14.8 ± 5.8) compared to patients with SSc with limited disease (8.3 ± 4.7) (*p* = 0.05), whereas there was no difference in macrophage infiltrate when comparing involved skin samples from patients with dSSc (27.2 ± 7.8) to skin samples from patients with lSSc (22.3 ± 11.7) (*p* = 0.78). The number of CD68^+^ cells was comparable in involved skin from patients with SSc despite anti-Scl-70 Ab positivity. In clinically uninvolved skin, patients with anti-Scl-70 Abs had a greater number of CD68^+^ cells (15.0 ± 5.1) than patients with SSc without anti-Scl-70 Abs (4.0 ± 1.4) (*p* = 0.02, Table 2).

### Cell infiltrate and skin score progression: the role of B cells

During follow up 12 patients were treated with rituximab with or without cyclophosphamide because of the progression of skin disease, while 16 patients did not receive any treatments because no progression of skin score was present at the time of biopsy (Table 3). At study entry, patients with SSc had comparable immunohistological findings in terms of CD68, CD3, CD20 and CD138 cells in clinically affected and unaffected skin samples after treatment stratification. However, treated patients had a decrease in skin score during follow up from 21.1 ± 10.9 to 11.2 ± 6.2 (*p* = 0.03), and all but one experienced a response >20% in the skin score. The clinical response in term of skin score improvement was not associated with the rate of baseline cell infiltrate.

**Table 3:** Clinical and histological characteristics according to immunosuppressive/immunomodulatory treatment and clinical outcome in the 28 patients with SSc

	Immunosuppressive treatment (12 patients)	No immunosuppressive treatment (16 patients)	*P*
Age (years)**,** mean (SD)	39.3 (13.9)	48.6 (16.2)	ns
Female/male, number	10/2	14/2	ns
Disease duration (months), mean (SD)	42.8 (100.3)	45.8 (41.5)	ns
Early/longstanding disease, number	10/2	9/7	ns
dSSc/lSSc number	11/1	9/7	0.05
Skin score baseline, mean (SD)	21.1 (10.9)	11.9 (10.6)	0.03
Anti-scl-70 antibodies positivity, number (%)	10	11	ns
ACA positivity, number	0	3	
ANA positivity, number	2	2	
Presence of CD20^+^ cell in skin biopsy, number (%)^a^	8 (66.7)	9 (56.3)	ns
CD20^+^ in clinically involved skin, mean (SD)	2.3 (3.1)	3.4 (6.3)	ns
CD20^+^ in clinically uninvolved skin, mean (SD)	1.5 (3.3)	0.9 (1.6)	ns
CD138^+^ in clinically involved skin, mean (SD)	3.5 (1.5)	3.8 (2.9)	ns
CD138^+^ in clinically uninvolved skin, mean (SD)	1.7 (1.8)	2.2 (2.1)	ns
CD3^+^ in clinically involved skin, mean (SD)	62.0 (28.9)	78.9 (37.4)	ns
CD3^+^ in clinically uninvolved skin, mean (SD)	43.7 (40.2)	47.2 (33.9)	ns
CD68^+^ in clinically involved skin, mean (SD)	25.3 (9.1)	38.0 (7.4)	ns
CD68^+^ in clinically uninvolved skin, mean (SD)	14.4 (5.7)	12.3 (6.9)	ns
Skin score after 6-month follow up, mean (SD)	11.3 (6.9)	13.0 (10.9)	ns
Patients with decrease >20% of skin score after 6-month follow up^b^	11 (91.7)	3 (18.7)	0.01
Patients with worsening >20% of skin score after 6-month follow up	0 (0.0)	7 (56.3)	0.01
*Patients with CD20 + cells on skin biopsy*	0 (0.0)	7 (77.8)	
*Patients without CD20+ cells on skin biopsy*	0 (0.0)	0 (0.0)	

The values are indicated as the mean (SD) or number (percentage) according to the data distribution

*SSc* systemic sclerosis, *ANA* antinuclear antibodies, *ACA* anticentromere antibodies, *anti-Scl-70* anti-topoisomerase antibodies, *dSSc* diffuse skin disease, *lSSc* limited skin disease, *ns* not significant

^a^Presence of at least one CD20+ cell in involved or uninvolved skin biopsy

^b^In this subgroup, six patients had CD20+ cells in uninvolved skin and were treated with immunosuppressive drug, while none of the untreated patients had CD20+ cells (*p* = 0.002)

Among the 16 patients that did not receive immunosuppressive therapy during the follow up, 9 patients had a stable skin score or worsening of the score was less than 20% after 6.5 ± 0.8 months, while 7 patients (43.7%) had more than 20% worsening of the skin score. The baseline and follow-up skin score in the latter patients were respectively 11.9 ± 10.6 and 15.7 ± 10.3 (*p* = 0.02). Among the 17 patients with B cell skin infiltrate at baseline, 8 patients were treated due to progressive skin involvement and 7 untreated patients had worsening during follow up. In the remaining two untreated patients with baseline B cell skin infiltrate, the skin score remained stable during the follow up.

In the 16 untreated patients, 9 patients with scleroderma (56.2%) had CD20^+^ cell skin infiltrate at baseline: 7 of them (77.8%) experienced worsening of the skin score, while none of the patients without CD20^+^ cells in the skin specimens experienced worsening of the skin score (Fisher’s test, *p* = 0.03). The mean number of CD20^+^ cells in involved skin from the seven patients with worsening of the skin score was higher than that in patients without worsening, but the difference was not statistically significant. Furthermore, the mean number of baseline CD138^+^ cells in involved skin (4.6 ± 3.6) and uninvolved (2.6 ± 1.1) skin biopsies was slightly higher in patients with skin progression compared to patients without skin worsening (involved skin 2.7 ± 1.1, uninvolved skin 1.7 ± 1.3), (*p* value not significant).

The mean number of baseline CD3^+^ cells in affected and unaffected skin of patients with a worsening of skin score (involved skin 79.3 ± 48.3, uninvolved skin 44.8 ± 37.0) was comparable to the mean number of CD3^+^ cells in patients with stable skin score (involved skin 78.6 ± 20.0, uninvolved skin 50.3 ± 31.9), (*p* value not significant in both comparisons).

Comparable results were obtained for CD68 staining; in fact, the mean number of baseline CD68^+^ cells in affected and unaffected skin in patients with skin progression (involved skin 31.3 ± 9.1, uninvolved skin 10.3 ± 4.7) was similar to the mean number of CD68^+^ cells in patients with a stable skin score (involved skin 24.7 ± 4.5, uninvolved skin 14.3 ± 9.3), (*p* value not significant in both comparisons).

## Discussion

A cutaneous cellular infiltrate is a frequent histopathologic finding in SSc [3, 6, 7, 10]. Cutaneous mononuclear cell infiltrates [3–12] may play a major role in starting and to mediating dermal sclerosis through their effects on fibroblasts [1].

In our study, T lymphocytes and macrophages were found in all cutaneous specimens from patients with SSc that were analyzed, predominantly in a perivascular location in the mid and/or deeper portion of the dermis. In previous studies, mononuclear cell infiltrates, including macrophages [3, 6–9] and T lymphocytes [3, 6, 8–12], with an increased number also of mast cells [3, 5, 6], were reported in SSc skin biopsies, above all in the early phases of the disease. In particular, Fleischmajer and co-workers [6] found that only 49% of patients with SSc had diffuse or perivascular cellular infiltrates in the dermis or subcutaneous tissue; the cells were identified as lymphocytes, plasma cells and macrophages. In this cohort, about 40% of the patients had early SSc and we did not find any associations with disease duration or immunological characteristics.

Evidence of cellular skin infiltrate has been reported previously in another study [10], in which up to 50% of the biopsies had relevant dermal mononuclear infiltration, but low/mild infiltrate was found in the other specimens [10]. The patients with relevant infiltrate frequently presented with diffuse disease, shorter disease duration [10, 11] and a higher skin thickening score [10]. The majority of T cells present in the skin infiltrate were activated T lymphocytes [10, 11], suggesting that early CD69^+^ T cells may actively participate in cell-cell contact with fibroblasts to induce fibrosis in skin lesions [11]. In our study, all specimens had relevant mononuclear infiltration. This discrepancy between the results of the previous studies [10] and our data may be due to differences in disease duration or in the area of the body from which the specimens were taken. Furthermore, genome-wide gene expression profiling studies of SSc skin biopsies have demonstrated wide heterogeneity, which can be quantitatively measured by DNA microarrays [13, 38–40]. Four specific gene expression signatures in both lesional and non-lesional skin biopsies have been identified in scleroderma compared to healthy controls [39].

Interestingly, in our study, the inflammatory cell infiltration was also present in the clinical uninvolved cutaneous specimens, in which the skin apparently did not have any clinical signs of thickness and sclerosis. This finding is in agreement with the indistinguishable pattern of gene expression found in clinically affected and clinically unaffected tissue from patients with SSc, which was clearly different from the gene expression in healthy subjects [13]. Our immunohistochemical data indicated that in patients with SSc the skin, even when considered as clinically uninvolved, is affected by an inflammatory process, suggesting that pathological changes can be detected even before the onset of skin sclerosis. Gene expression profiling studies have confirmed an altered signature in unaffected skin in patients with systemic sclerosis [13, 16], highlighting the truly systemic inflammatory nature of the disease. Interestingly, in our study, the number of CD68^+^, CD3^+^, CD20^+^ and CD138^+^ cells was higher in involved skin than in uninvolved skin, suggesting a local role for these cells in accelerating the fibrogenic processes, that lead to clinical skin modifications. The role of macrophages in different target organs in patients with SSc has been recently demonstrated [15, 41] and confirmed in multiple cohorts with skin disease, showing that the gene expression of several activated macrophage markers are elevated in the skin in SSc [14]. Activated macrophages likely play a pivotal role in the pathogenesis of SSc by activating fibroblasts, but as these cells are plastic and readily modulated by the local tissue microenvironment, the cytokine milieu determined also by the presence of B and T lymphocytes, dendritic cells, endothelial cells and fibroblasts might play a role in the pathogenesis of the fibrotic process in the skin [41].

The role of T cells in SSc is already accepted [42]. In this regard, all SSc specimens examined in our study had significant CD3^+^ cell infiltrate mainly in clinically involved but also in uninvolved skin, supporting the concept that the dermal T lymphocytes play a key role in local pathogenic events in SSc by cytokine production and that lymphocyte/fibroblast interaction in the skin is important in the pathophysiology of SSc. However, the main clue to the specific importance of the immune cell infiltrates did arise from the longitudinal assessment of the course of the skin involvement. The mechanistic exploration of the pathogenic infiltrate still needs to be defined. The positive response to rituximab in a subgroup of patients with SSc, regardless of the baseline presence of B cell skin infiltrates, indirectly supports the notion that there might be a complex relationship among all mononuclear cells in determining clinical modification and fibrosis in scleroderma and probably the T and B cell crosstalk can have a role in promoting the fibrotic process.

Even though a recent analysis of DNA microarrays of cutaneous biopsies from patients with dSSc demonstrated higher expression of clusters of genes in CD20^+^ cells [13], in some immunohistochemical studies the B cells were rare or absent [3, 8–10]. Recently, the analysis of B cell infiltrate performed in skin biopsies from patients treated with anti-CD20 therapy demonstrated improvement in the skin score and stabilization of FVC, DLCO and clinical symptoms [17–21, 23]. In our study, B cell infiltrate seems to be characteristic of the skin in scleroderma, in fact CD20^+^ and CD138^+^ cells were absent in all specimens from healthy subjects. B lymphocytes were detected in up to 61% of patients with SSc and the mean number of CD20^+^ and CD138^+^ cells was lower than the number of CD3^+^ and CD68^+^ cells. Fifty percent of these patients had B cells both in involved and in uninvolved skin, while the other 50% had B cells only in involved skin, while plasma cells were present in all involved skin and in all but three samples of clinically uninvolved skin. The presence of plasma cells suggests that specific chemotaxis or local maturation of memory B cells had occurred. The wide variability in the identification of B cells in skin biopsies in our study and in other studies could be related to the different cell signatures previously reported [39]. Certainly, the clinical effects seem not to depend upon the cell numbers found in the skin biopsy samples.

The great majority of patients with SSc who had CD20^+^ cells on skin biopsies had anti-Scl-70 Ab positivity or early disease or diffuse skin involvement*.* Patients with early disease had a greater number of CD20^+^ cells in involved skin compared with patients with long-standing disease, and the presence of CD20^+^ cells in uninvolved skin seems to be characteristic of patients with dSSc. Our sample size was not large enough to draw definite conclusions, but our data suggest that B cells are associated with the classical negative prognostic factor of scleroderma such as diffuse phenotype and antiscl-70 Ab positivity. Among the various immune cells, B cells appear to be the only ones linked to progression of skin involvement over time.

Albeit the number of patients enrolled in this study was limited, the worsening of skin involvement in the group of patients with CD20^+^ cells on skin biopsy after 6-month follow up suggests a possible prognostic value of B cell presence in the progression of skin involvement. No prognostic role was evident for plasma cells, suggesting that very likely they were already there, and that the arrival of the new B cells was the most important event for the diseased skin. The presence of B cells in the majority, but not in all patients, indicates an important, though not essential role for cutaneous B cells in more aggressive scleroderma disease, above all during the earliest phases when the inflammatory process and cell-cell interactions very likely play an essential role in the development of fibrosis over time. B cells in the skin may be involved in the initiation and expansion of the SSc inflammatory process. Of interest, even plasma cells were present either in the involved or the uninvolved skin and in 10 patients in this cohort on analysis of the peripheral blood B cell subsets*,* we found that the number of CD20^+^ and CD138^+^ cells in the skin specimens correlated directly with the percentage of circulating CD27^+^CD38^+^ plasmablasts (unpublished data). Either resident and circulating B cells can determine, with their multiple functions as Ab-producing cells, antigen presenting cells and profibrotic and proinflammatory cytokine (IL-6, IL-4, transforming growth factor (TGF)-β)-secreting cells [1, 43], an autoimmune milieu that could be of great impact in the development of fibrosis. B cells directly stimulate fibroblasts by a direct contact-based mechanism [44] and a plethora of soluble factors present in the inflammatory skin condition contributes to the commitment of B cells in autoantibody-producing plasma cells, which, again, can directly stimulate fibroblasts.

The limited number of patients with scleroderma and of healthy controls in this study did not allow us to infer robust correlation with disease phenotype and organ involvement, but further studies on sequential biopsies after therapy could allow us to investigate temporal associations with different types of cell infiltrates, and to test correlation with the characteristics of inflammatory infiltrate with fibrotic involvement, such as the collagen score and myofibroblast score.

## Conclusions

These results highlight that a subgroup of patients with SSc exhibit an imbalance in B cell infiltrate and that cutaneous mononuclear cells of the innate and adaptive immune system may play a role in mediating dermal fibrosis in different stages of scleroderma disease and in patients with diffuse skin involvement. Therapeutic approaches decreasing the numbers of cutaneous lymphocytes, in particular B cells, and/or interfering with their functions might prove useful in the management of cutaneous involvement in SSc from the earliest phases of the disease.

## Additional file

Additional file 1: **Table S1**. Autoantibody characteristics according to scleroderma skin disease extension. (DOCX 14 kb)

## Abbreviations

Abs: Antibodies
ACA: Anti-centromere antibodies
ANA: Antinuclear antibodies
anti-Scl-70 Abs: Anti-topoisomerase antibodies
DLCO: diffusing capacity for carbon monoxide
dSSc: Diffuse systemic sclerosis
ELISA: Enzyme-linked immunosorbent assay
ESR: Erythrocyte sedimentation rate
FVC: Forced vital capacity
GH: Global Health
H_2_O_2_: Hydrogen peroxide
HAQ: Health Assessment Questionnaire
HRCT: High resolution computed tomography
IL: Interleukin
lSSc: Limited systemic sclerosis
LVEF: Left ventricular ejection fraction
mAb: monoclonal antibodies
PASP: Pulmonary artery systolic pressure
PFT: Pulmonary function test
RT: Room temperature
SD: Standard deviation
SSc: Systemic sclerosis
TBS: Tris-buffered saline
