# Supplementary material for: Characterization of inflammatory cell infiltrate of scleroderma skin: B cells and skin score progression
Source: Arthritis Res Ther. 2018 Apr 18;20:75. doi: 10.1186/s13075-018-1569-0 (PMC5907298; doi:10.1186/s13075-018-1569-0)
Supplement: Supplementary file 1 — Table S1. Autoantibody characteristics according to scleroderma skin disease extension. (DOCX 14 kb) [file 13075_2018_1569_MOESM1_ESM.docx]

**Additional file 1: Table S1. Autoantibodies characteristics according with scleroderma skin disease extension.**

|  | Diffuse skin disease  20 pts | Limited skin disease  8 pts |
| --- | --- | --- |
| Antiscl-70+ patients | 17 (85.0) | 4 (50.0) |
| Anticentromere+ patients | 0 (0.0) | 3 (37.5) |
| RNA polymerase III patient | 1(5.0) | 0 (0.0) |
| ANA posivive patients | 2 (10.0) | 1 (12.5) |
